# Orthoplastic Reconstruction of Distal Tibia High-Energy Fractures Using a Circular External Fixator—A Systematic Review

**DOI:** 10.3390/jcm13195700

**Published:** 2024-09-25

**Authors:** Radu-Dan Necula, Simona Grigorescu, Bogdan-Radu Necula

**Affiliations:** Faculty of Medicine, “Transilvania” University, 500036 Brasov, Romania; radu.necula@unitbv.ro (R.-D.N.); simona.grigorescu@unitbv.ro (S.G.)

**Keywords:** Ilizarov, open fracture, lower limb, flap, reconstruction

## Abstract

**Background**: Compound fractures of the distal tibia (with or without the middle third) represent a challenge for orthopedic and plastic surgeons because of the scarcity of available soft tissue reconstruction and the important comminution of the fractures that usually appear. **Methods**: The design of this study is based on the PRISMA guidelines. Databases were searched for articles published and available until the first half of 2023. Articles that presented the evolution of patients treated by combining circular external fixators with reconstructive methods were selected. **Results**: After searching the literature using keywords, we obtained 3355 articles, out of which 14 articles met all the inclusion criteria, with a total number of participants of 283. The bone loss varied between 0.7 and 18.2 cm, while the soft tissue defect was between 3/3 cm and 16/21 cm. The average period of fixation ranged from 4 to 22.74 months. The most used reconstruction methods were 80 free flaps and 73 pedicled flaps out of 249 interventions. Complete flap loss appeared only in 3/283 patients. Regarding the bone union, the percentage of non-union was low, and in all cases, it was achieved after reintervention. A low rate of major complications was observed. **Conclusions**: The orthoplastic team is the key to successfully treating the high-energy traumatism of the distal tibia (with or without a middle third). The Ilizarov external fixator can be used as a definitive limb-salvage treatment (secondary to the standard primary methods of fixation) in combination with a flap to cover the defects because it does not damage the pedicle, and it helps stabilize the soft tissues and bones around the flap to lower the complications.

## 1. Introduction

Distal tibia fractures represent one of the most common causes of visiting the emergency room necessitating orthopedic treatment. The incidence of the distal tibia fracture is the second most seen fracture of the tibia after the middle third of the tibia [1,2,3]. The incidence of this type of fracture fluctuates between 71 and 179 cases per 100,000 person-years in different studies [4,5].

Even though compound fractures of the distal tibia are rare, in the high-energy traumatism compound, the fractures of the distal third (with or without the middle third) of the tibia are more frequent. The AO classifications of these types of fractures are either 43C2.3 (complete, simple articular, multifragmentary metaphyseal fracture extending into the diaphysis) or 43A3.3 (extra-articular, multifragmentary fracture extending into the diaphysis) [6]. In these cases, the treatment should be adapted to both segments (distal and middle third). Complex limb-salvage procedures or amputation are the two options available to the team (orthopedic and plastic surgeon) in these situations [7]. One of the most feared complications is iatrogenic damage of the soft tissue. The risk is greater if the fracture is classified as a Gustilo–Anderson type III [8].

Orthopedic surgeons have to face an important choice of how to manage the fracture so that the soft tissue will not be affected further. As is known, the open reduction internal fixation (ORIF) approach necessitates prolonging the incision followed by extensive dissection, which are acts that have a high chance of disrupting the frail vascularization of the suffering soft tissue [9]. The threat of further damage to an already suffering tissue should be minimized; there are review papers that support the usage of an external fixator (Ex-fix.) as a final method of fixation for open tibial fractures [10]. The circular Ex-fix. developed by G. Ilizarov (and other newer versions of it) is known for the low impact it has on soft tissue. It is proposed as a suitable option in these particular fractures because of the stability of the frame, versatility of construction, and minimally invasive technique [11]. On the other hand, Daniels et al. showed in their meta-analysis that patients in the Ex-fix. group may develop more complications than patients in the ORIF group [12].

Regarding the soft tissue damage, the reconstructive options at this level are scarce and require a well-prepared surgeon [13]. The plastic surgeon has to take into account the patient’s comorbidities, the fixation options for the fracture, the quality of the local soft tissue, and vascularization. The options may vary from skin grafting to free-flap transfer. Amputation may be an option if the local damage is so extensive that the function is dramatically affected [14,15]. This type of injury needs to be treated by an orthoplastic team, a term introduced in 1993 by Scott Levin [16]. Beginning in 2017, the United Kingdom introduced orthoplastic care as the standard of care for open fractures of long bones [17].

Unfortunately, in cases of extensive bone damage (affecting both the middle and the distal third of the tibia), there is a lack of consensus regarding the optimal fixation method. Ex-fix. has been observed to have a higher malunion rate compared to intramedullary nailing. Although in fractures associating important bone loss, the circular Ex-fix. may be preferred, but there is no clear indication [7]. Furthermore, there is a lack of studies recommending the optimal method of fixation in combination with a reconstructive technique. In this review paper, the aim is to cover the data known about the orthoplastic treatment of the compound fractures of the distal and middle tibia by describing and highlighting the advantages and disadvantages of the circular Ex-fix. along with a reconstructive technique as a definitive treatment for open distal and middle tibial fractures.

## 2. Materials and Methods

This paper was designed as a systematic review, and this review has been reported conforming to the Preferred Reporting Items for Systematic Reviews and Meta-analyses (PRISMA) reporting guidelines [18]. In order to write the present review of the literature, it was not necessary to obtain the approval of the Ethics Committee. The protocol for this review has not been registered.

### 2.1. Search Strategy

Two researchers (B.N. and R.N.) searched databases (PubMed; Springer; Science Direct) during the year 2023 by applying the following keyword search: Ilizarov, ankle, flap, external fixator, complex, lower limb. The keywords were combined using the connectors OR and AND in groups of three, applied to each database at a time. The selected articles had to be published and available until May 2023. Both researchers (B.N. and R.N.) performed the screening of the databases independently by reading the title and the abstract of the papers and eventually selecting the ones appropriate for the review. During the next step, while reading the entire text, the two reviewers applied the inclusion and exclusion criteria presented below. In the end, duplicate articles were removed.

The inclusion criteria used during the reading of full-text research papers were as follows: (1) compound fracture of the distal third of tibia (with or without middle third); (2) circular Ex-fix. with a reconstructive technique; (3) patients over 15 years old; and (4) reporting the evolution of the patient and complications.

On the other hand, the exclusion criteria applied were as follows: (1) treatment for chronic pathology; (2) another language than English; (3) data not clear about the type of fixation; (4) no data/few details about the evolution of the patients; (5) the full-text was not available; (6) review articles; and (7) proximal tibia fracture.

### 2.2. Data Selected

The relevant data were selected by two reviewers (B.N. and R.N.) after generating the final list of articles. The following information was extracted from the papers: first author; year; period of study; country; number of participants; mean age of the participants; type of fracture; soft tissue defect size; bone defect size; period of fixation; type of reconstructive surgery; outcomes; and complications.

The primary outcomes of this study are the rate of achievement of bone union, soft tissue reconstruction success, return to work, and ambulation ability.

### 2.3. Quality Assessment

In order to quantify the risk of bias and the quality of the selected studies, the two reviewers (B.N. and R.N.) calculated the Methodological Index for Non-randomized Studies (MINORS) [19]. If there were disagreements, they were resolved by discussion and by reaching a consensus.

## 3. Results

**Figure 1:**
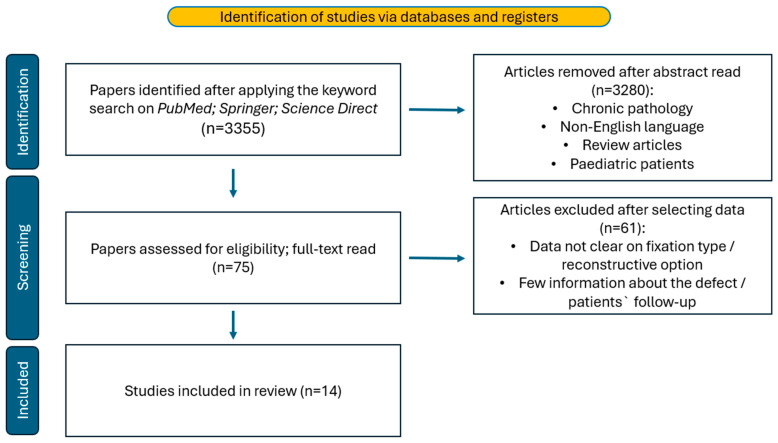
The flow-diagram of the literature search.

After searching the database using the keywords, we obtained 3355 articles. At the end of the screening and full-text reading, 14 articles met all the criteria. The detailed search methodology is shown in Figure 1. After a full data review, the authors decided to perform a qualitative analysis. This choice had to be made because of the data being assessed in different ways between the articles. The detailed presentation of the articles can be seen in Table 1.

### 3.1. Quality Assessment–Results

Applying the Methodological Index for Non-randomized Studies survey, the risk of bias was evaluated. For noncomparative studies, the highest score would be 16, while for comparative studies, the highest score would be 24 [19]. The detailed scores are presented in Table 2 (MINORS).

### 3.2. Articles Characteristics

Of the 14 studies included in this review, 12 are non-comparative, while 2 are comparative. The total number of participants across these studies is 283, with a mean age of 39.01 years. The articles were published between 1995 and 2022, with a notable trend indicating that most of the studies were published after 2015. This trend is also reflected in the selected articles for this review, the majority of which were published in 2017 or later. There is a growing interest in the combination of external circular fixation and reconstructive techniques, leading to an increase in research activity in this area each year.

Regarding the country where the studies have been conducted, five out of fourteen were completed in China. Additionally, it is noteworthy that over 50% of the research has been carried out in the eastern region of the world. This prevalence may be attributed to the increased popularity of Ilizarov, and his technique has been more popular in countries such as Russia, China, Eastern Europe, etc.

The number of participants in each study is low. Yongwei Wu et al. managed to introduce 50 patients in his study, in contrast with Abulaiti Abula et al., who had a cohort of only 18 patients [27,29]. This type of fracture typically occurs after a high-energy traumatism, resulting in an open fracture and soft tissue defect. As seen in studies, these cases do not present often in the Emergency Room (low number of patients in the selected articles), but when they do, they require an extended duration of treatment and complex techniques.

For all the participants, the circular external fixator was employed as a definitive method of osteosynthesis; however, it was not employed as the initial fixation method. As the first step, a mono-planar or bi-planar external fixator was applied following thorough debridement, applying the standard method of acute care in open fractures.

The indication for implementation of the circular Ex-fix. has been stated in three papers. Two studies used the circular Ex-fix. as an alternative and last method of treatment before amputation [24,33]. Jones et al. suggested that this technique should be used in high-energy fractures accompanied by significant soft tissue defects, as it facilitates closure and moving down the reconstructive ladder [32].

### 3.3. Bone Injury Results

The patients enrolled in the articles selected for this review had a complex traumatism which led to the fracture of the distal tibia and to the injury of the soft tissue. All of them were treated using a type of circular external fixator as a method for fixation for an acute fracture. The majority of the cases were initially classified as Gustilo–Anderson (GA) type III B at the first look—Table 3. Although the selected papers report patients classified as GA type I or II, because of the local and external factors observed during the second evaluation, these patients have been considered GA type III. In the study run by David W. Lowenberg et al., there were a few patients who initially presented to the ER with a closed fracture, but because of the injury of the local vessels, the soft tissue became necrotic and the fracture has been managed as a compound fracture [24].

In almost all cases (12/14), a certain degree of bone loss has been described. This phenomenon can be attributed to two primary factors. The first factor is the destruction of the bone as a result of trauma. The second factor pertains to the nature of the fractures documented in the selected studies, which are characterized not only as open fractures but also as comminuted fractures., Such conditions have led to a diminished blood supply to the bone at the site of injury. Moreover, because of the open wound, the fractures can be described as being contaminated, so after thorough debridement, a bone defect has appeared. The bone loss has been observed to range from 0.7 to5.3 cm in Christine M. Jones et al., and from 8 to 18.2 cm in the paper by Yong-Qing Xu et al. [31,32]. In the majority of cases, the bone defect has been treated either by using the distraction and bone lengthening method or by bone transport, as detailed in Table 4. Bone grafting has also been used, but almost exclusively as an adjuvant treatment for non-union or delayed union.

The average period of fixation ranged from 4 months to 22.74 months. Regarding the lengthening, in almost all studies, the patients presented a small limb length discrepancy (LLD) (26 out of 283 patients), with most of the patients having LLD under 2 cm.

### 3.4. Soft Tissue Damage

In all selected cases, soft tissue injury was observed; however, only nine out of fourteen reviewed studies provided measurements of the defects. The largest range of dimensions is in the research of Yongwei Wu et al., varying from 3/3 cm to 16/21 cm. All of the defects necessitated a method of reconstruction, as presented in Table 5.

Because of the local anatomy and the scarcity of tissue surrounding the bones and tendons in the described area, it is necessary to use a more advanced technique, a higher step on the reconstructive ladder [15]. This observation is corroborated by the articles included the review. Among 249 interventions aimed at repairing the defect, 80 have been free flaps, while 73 have been pedicled flaps. The most frequently used as a free flap has been the Latissimus Dorsi muscle and the most used pedicled flaps have been the sural neurovascular flap and the great saphenous neurocutaneous flap. On the opposite side, the split-thickness skin graft (STSG) (7.22%) and local flaps (3.61%) have been rarely used. Some other methods have also been tried, such as in the study of G. Hosny et al., who investigated the use of the skin traction using K-wires attached to the skin [22].

### 3.5. End Points and Complications

The primary endpoints of the studies are bone union and flap survival, as presented in Table 6. Regarding bone union, it has been achieved in all cases. The incidence of non-union is low, at 18.37% (52 patients); however, it is noteworthy that bone union was ultimately attained in all cases following reintervention.

Regarding the flap survival, the rate of survival is over 90% in almost all studies (83.33%). Complete flap loss occurred in only three out of two hundred and eighty-three patients (1.06%). In the other cases, it has been a partial tip necrosis which, except for one patient, has been treated by regular dressings.

The main aim of this technique is to prevent amputation and to salvage the lower limb. Out of a total of 283 cases, only 1 required amputation; however, this was not for bone non-union or flap damage. The reason for amputation was the patient’s chronic pain in the ankle at the end of the treatment [30].

Complications have been categorized by various authors as major or minor. Because of the lack of exhaustive data in certain papers, we have presented them as a single table, Table 7. Most of the complications (69 patients out of 283–24.38%) are superficial infections at the level of the pin insertion. These infections can typically be managed through the application of dressings and by administering antibiotics. On the other hand, there have been more serious complications that necessitated additional surgical intervention. Among these complications are malunion (4.94%), joint stiffness (10.95%), osteomyelitis (2.47%), etc.

The pain may be one of the most important complications associated with circular Ex-fix. treatment. In a cohort of 27 patients (9.54%), individuals reported experiencing pain during or following the treatment. Among these patients, 14 exhibited severe pain that persisted after the removal of the fixator, necessitating chronic pain management interventions. Some of them changed their workplace and reduced their daily physical activity, while one of them required the amputation of a limb. The other 13 had mild to moderate pain during the treatment and which was effectively managed either by pausing the distraction of the apparatus for a week or with analgesic treatment.

Regarding the evolution of the patients, 124 patients required reintervention for non-union, refracture, or malalignment. Out of those, 92 patients (74.2%) have been managed by reapplying the circular Ex-fix. and if necessary (non-union cases), in combination with bone graft. The remaining 32 patients (25.81%) were treated by switching the method of fixation from circular Ex-fix. to ORIF.

Limb length discrepancy was observed in only 26 out of 283 cases, indicating the feasibility of using this technique in reconstructing bone defects. Out of those cases, 13 patients (4.6%) had a limb length discrepancy greater than 2 cm [21].

Moreover, we have observed the percentage of patients who regain normal walking without braces or crutches. This endpoint has been described in seven out of fourteen papers (as described in Table 1). A total of 76 out of 86 patients included in the analysis have successfully regained normal walking, resulting in a percentage of 88.37%.

An important complication associated with the treatment of bone loss in the middle and distal third of tibia is the potential development of muscle and Achilles tendon contractures. Muscle and Achilles tendon contractures have been described in three included studies [23,29,31]. In two of these studies, except for the findings reported by Hasankhani et al., the patients eventually regained the normal function of the ankle joint after physiotherapy, subcutaneous Achilles tendon lengthening, or the application of an external fixator. In these instances, the external fixator was utilized as a therapeutic approach to address complications arising during treatment [29,31]. On the other hand, contrary to what has been described previously, Hasankhani et al. observed that 10 patients (out of 32–31.25%) presented a noticeable limp, and 3 patients did not return to work [23].

Joint stiffness after treatment is a feared complication. In a cohort of 31 patients, 10.95% exhibited joint stiffness and restricted mobility in either the ankle or knee [22,23,25,31,33]. Subsequent physiotherapy resulted in a notable reduction in joint stiffness, enabling 19 out of the 31 patients (61.29%) to ambulate normally without the assistance of braces or crutches. Moreover, Lim et al. reported the presence of ankle post-traumatic arthritis in 5 patients (GA III A and III B) out of 11 patients in the GA III group [30].

In relation to the scoring systems employed for the evaluation of outcomes, three studies reported results based on the Association for the Study and Application of the Method of Ilizarov (ASAMI) classification [23,29,31]. The majority of the patients presented with either excellent or good bone and functional results. Another classification used to assess the patient’s evolution has been Paley’s Evaluation of Bone and Functional Results [27,33]. Notably, over 70% of patients demonstrated excellent bone results, and similarly, over 70% achieved excellent or good functional outcomes, as illustrated in Table 8.

## 4. Discussion

The type of injury previously discussed is associated with high morbidity for the patient and significant costs for the health provider. The decision to salvage or amputate a mangled extremity remains a contentious issue; a Gustilo type III (B and C) injury may necessitate amputation despite attempts at reconstruction due to the challenges posed by irreparable damage or vascular compromise [34]. In this study, most of the patients have been classified as a Gustilo type III (84.1%), which implies that the soft tissue and bone are extremely damaged. In the cases categorized as GA type I and II groups, because of the local complications such as infection or necrosis, along with extensive debridement, resulted in defect sizes that the patients were assigned in the Gustilo type III group. The reason for amputation in those cases is correlated with the accessibility to methods of reconstruction and the complications which may appear afterwards. In our review, limb salvage was achieved in all but one case, where amputation was performed due to persistent residual pain [30]. Although failure to reconstruct the lower limb and to allow the patient to regain a normal life has appeared in only one case, it should be noted that there might have been multiple reasons for this. It is noteworthy that the necessity for amputation was not attributable to the failure of fixation methods or the reconstructive techniques. Furthermore, regarding the functional results, more than 70% of all the patients for whom a score was calculated (142 patients) have presented a value of at least good.

The reconstructive options in distal tibia soft tissue defects are limited. The goal is to cover the defect using well-vascularized tissue while stabilizing the fracture [35]. Advances in plastic surgery techniques have facilitated an increased utilization of free tissue transfer, thereby enabling limb salvage in cases where amputation would have historically been the sole option [36]. In our observations, the predominant methods for covering the soft tissue defect have been the free flap transfer (80 cases out of 283) and pedicled flaps (73 out of 283). Consistent with the study of Kozusko et al., one of the most used free flaps to perform the coverage in distal tibia reconstruction has been the Latissimus Dorsi flap [15]. Among the pedicled flaps, the Gastrocnemius flap and the Sural neurocutaneous flap have been the most commonly applied. Notably, only eight cases (out of two hundred and thirty) have been reported in which the flap did not survive, either partially or completely. In these instances, further treatment was required, predominantly conservative management for cases of partial necrosis, with only three cases necessitating an additional flap procedure. The choice of the flap has been made based on the local vascularization, position, and surface of the defect. Bekara et al. described that the survival of free flaps is comparable with those of pedicled flaps and the success of coverage is similar between the techniques [37]. Consequently, the choice of flap is contingent upon the specific location of the defect, local anatomical considerations, and the surgeon’s experience. Additionally, selected studies have also described other methods of reconstructing the soft tissues of the distal lower limb. Hosny et al. used skin traction (four patients) by modifying the apparatus so that K-wires were applied to the skin to pull it over the defect [22]. Negative pressure wound therapy has been used as a bridge to permanent coverage of the defect [30]. Xu et al. used soft tissue transport in parallel with bone transport, with the residual soft tissue defect subsequently repaired using split-thickness skin grafting [31].

Both reconstructive surgery and bone fixation were performed either concurrently or in closely sequenced procedures. As previously noted, the survival rate of the flap was 83.33%, indicating that the combination of reconstructive surgery and the application of Ilizarov’s external fixator is effective for the management of high-energy traumatism to the distal tibia. The use of Kirschner wires, which possess a small diameter, minimizes disruption to the vascularization of the surrounding soft tissue. Because of this, the covering tissue is not affected by the osteosynthesis method [26].

The combination of orthopedic surgery and plastic surgery has managed to save the intensely traumatized lower limbs. The external circular fixator is known to be useful in the limb salvation of infected non-unions of the lower limbs. As Khan et al. described in 2015, despite the challenges posed by infection, the surgical team successfully achieved bone union, thereby facilitating the restoration of function in the affected patients. The Ilizarov apparatus has the great advantage of the possibility of performing compression osteogenesis, distraction osteogenesis, or bone transport which will support bone healing and reduce limb-length discrepancy [38,39]. In this review, it was observed that 104 patients out of 283 reported infections prior to the application of the Ilizarov external fixator; in all instances, the infections were effectively managed while achieving bone union. Because of its characteristics, this type of fixation is a suitable method of treatment in such cases.

The opportunity of having bone regeneration without needing a bone graft as a first option is one of the reasons why an orthopedic surgeon will choose this technique for treating fractures with bone defects [11]. In this review, compression distraction osteogenesis after bone shortening was performed in 63 cases, while bone transport was performed in 142 cases—Table 6. Bone grafting was considered as a treatment option in 56 cases, predominantly as a strategy for addressing non-union or delayed union. Our findings are consistent with the observations made by Papakostidis C et al., who noted that bone grafting has been used mainly at the docking site, as an adjuvant for bone regeneration in the case of delayed union [40]. Ultimately, bone union was achieved in all cases.

It has been observed that the use of open reduction and internal fixation (ORIF) in high-energy fractures of the distal is associated with a significant rate of complications, predominantly involving soft tissue. In contrast, the Ilizarov external fixator demonstrates a lower incidence of complications; however, it may not be appropriate for intra-articular fractures [41,42]. In this review, the most common complication has been superficial pin-tract infections which have been treated conservatively. Malunion has been observed in 4.94% of the cases, while joint stiffness (knee or ankle) has appeared in 10.95% of cases. Pain has been described as a complication in 9.54% of cases, although it has significantly impacted the quality of life in only one instance. [30] The overall complication rate is comparable to those reported the ones described in other conditions treated with circular external fixation, indicating that the majority of complications can be classified as minor (pin-site infection or mild pain). Conversely, a minority of cases (less than 5%) have been categorized as significant complications, including malunions, refractures, and osteomyelitis [43,44,45].

## 5. Limitations

One of the most important limitations of this study has been the diversity of scores through which the results have been quantified. The variety of scores utilized has hindered our ability to obtain a clear representation of the findings. The most used scores in other articles which evaluated the usage of the Ilizarov Ex-fix. in lower limb pathologies have been the Association for the Study and Application of Methods of Ilizarov (ASAMI) and also Paley’s Evaluation of Bone and Functional Results [46,47]. The authors consider that the limb salvage utilized as an end point is useful but the lack of score assessment has hindered our ability to obtain a clear representation of the findings and the provision of a more comprehensive understanding of the outcomes.

An additional significant factor that is often overlooked is the duration of hospitalization. This parameter can provide a comprehensive understanding of the challenges associated with managing such complex cases and what impact they have on the health system.

## 6. Conclusions

The orthoplastic team plays a crucial role in the management of high-energy trauma to the distal tibia, particularly in cases where the soft tissue availability is limited and bone fractures may compromise the functionality of the lower limb. The Ilizarov external fixator, along with other comparable techniques, offers significant advantages in the stabilization of bone fragments while minimizing soft tissue injury because of the small diameter of the K-wires which are inserted in a minimally invasive manner. Moreover, as has been seen previously, the Ex-fix. can be used in combination with a flap to cover the defects because it does not damage the pedicle while protecting the soft tissues (low iatrogenic traumatic effect) around the flap, offers the best rigidity of the available Ex-fix. types at the fracture level, and can be personalized for each patient (3D design).

## Figures and Tables

**Table 1:** Study characteristics. NM, not mentioned.

Author	Published Year	Period of Study	Country	Number of Participants	Mean Age	Type of Fracture	Soft-Tissue Defect Size	Bone Defect Size	Mean Time of Fixation	Type of Reconstructive Surgery	Outcomes	Complications
D.S. Jorgenson [20]	1995	1992–1993	USA	3	32.33	GA III	NM	>8 cm	41.67 weeks	Free flaps (gracilis)/gastrocnemius flap	Union rate/Flap survival/Functional recovery/ambulatory status	Free Flap loss/Pseudoarthrosis
H.S. Kim [21]	1998	1997	Korea	1	20	GA III	10/20 cm	17 cm	NM	Latissimus Dorsi muscle free flap	Union rate/Flap survival/Functional recovery/ambulatory status	Gradual transportation failure/Limb-length discrepancy
G. Hosny [22]	2003	1993–1999	Egypt	30	33.1	GA I/II/III	NM	4–15 cm	11.1 months	split thickness skin graft/Z-plasty/pedicled flaps	Union rate/angular deformity	Deep venous thrombosis/eczema/pin-tract infection/malunion/
Ebrahim Hasankhani [23]	2006	1999–2002	Iran	32	32	GA III	NM	3–9 cm	21 weeks	split thickness skin graft/fasciocutaneous flap/gastrocnemius or soleus flaps/free flaps	ASAMI classification	Pin-tract infection/Residual deformity/Shortening more than 2.5 cm/stiff joint/significant pain
David W. Lowenberg [24]	2013	1993–2005	USA	34	40	closed/GA II/GA III	88 cm^2^	8.7 cm	10.8 months	free flaps (rectus abdominis; latissimus muscle flaps)/rotational gastrocnemius flaps	Union rate/reoperations/ambulation status/working status	Flap loss/non-union/malunion/infection/chronic pain/refracture
J.P. Repo [25]	2016	1989–2014	Finland	16	33	GA III	4/6 cm to 10/30 cm	4.5–12 cm	178 days	microvascular Latissimus Dorsi muscle/musculocutaneous flap	DASH score/FIT index/HRQoL score/Lower Extremity Functional Scale	Severe pain/limited joint mobility/partial flap necrosis/Osteomyelitis/Pseudoarthrosis/refracture/malunion/delayed union/pin-site infection
Jia Xu [26]	2017	2007–2012	China	18	41.2	compound fracture ankle	8/9 cm to 14/18 cm	2–6 cm	11.4 months	great saphenous neurocutaneous flaps/peroneal perforator-based sural neurocutaneous flaps	Flap survival/union rate/range of motion/bone alignment	Pin-tract infections/developed mild contracture of the Achilles tendon/pain/non-union
Yongwei Wu [27]	2017	2007–2016	China	40	39	GA II/GA III	3/3 cm to 16/21 cm	4.3–11.0 cm	NM	local fasciocutaneous flap/split thickness skin graft	Union rate/functional recovery	Pin-tract infections/limb length discrepancy/pin-tract loosening/neurological damage/pain
Mitsuhiko Takahashi [28]	2020	2020	Japan	1	70	GA III	15/20 cm	NM	4 month	Latissimus Dorsi muscle flap	Lower Extremity Functional Scale/the timed up and go test	NM
Abulaiti Abula [29]	2020	2010–2017	China	14	35.5	GA III	20–54 cm^2^	4–12.5 cm	208.5 days	Posterior tibial artery perforator flap/sural neurocutaneous flaps/Latissimus Dorsi flap/anterolateral thigh flap	Union rate/ambulation status/functional assessment	Partial tip necrosis of the flap/pin-tract infection/muscle contraction/axial deviation
Jiang An Lim [30]	2020	2014–2019	UK	20	50.45	GA I/II/III	NM	NM	10.4 months	radial forearm free flap/negative pressure wound therapy	AOFAS score	Post-traumatic arthritis/delayed union/deep infection/pin-tract infection/pulmonary emboly/severe pain/Acute Compartment Syndrome
Yong-Qing Xu [31]	2021	2009–2016	China	31	33.4	GA III	56–288 cm^2^	8–18.2 cm	22.74 months	primary suture–soft tissue transport/split thickness skin graft	ASAMI classification/Paley’s Evaluation of Bone and Functional Results	Muscle contraction/local infection/delayed union/non-union/joint stiffness/refracture
Christine M. Jones [32]	2021	2005–2020	USA	18	47	GA III	NM	0.7–5.3 cm	NM	split thickness skin graft/local fasciocutaneous flap/pedicled muscle flaps/free tissue transfer	Union rate/ambulation/flap survival	Leg length discrepancy/Partial loss of flap or graft/Flap loss/
Yuan-Jian Wu [33]	2022	2010–2016	China	25	39.2	GA III	10/5 to 14/12 cm	4.5–9.5 cm (6.94 cm)	NM	sural neurovascular flap/greater saphenous neurocutaneous perforator flap	Paley’s Evaluation of Bone and Functional Results	Pain/pin-tract infection/mild ankle midfoot joint stiffness/non-union

**Table 2:** MINORS scores—detailed.

MINORS Score Criteria	D.S. Jorgenson, 1995, [20]	H.S. Kim, 1998, [21]	G. Hosny, 2003 [22]	Ebrahim Hasankhani, 2006 [23]	David W. Lowenberg, 2013 [24]	J.P. Repo, 2016 [25]	Jia Xu, 2017 [26]	Yongwei Wu, 2017 [27]	Mitsuhiko Takahashi, 2020 [28]	Abulaiti Abula, 2020 [29]	Jiang An Lim, 2020 [30]	Yong-Qing Xu, 2021 [31]	Christine M. Jones, 2021 [32]	Yuan-Jian Wu, 2022 [33]
Clearly stated aim	2	1	2	2	2	2	2	2	1	2	2	2	2	2
Inclusion of consecutive patients	2	1	2	2	2	2	2	2	1	2	2	1	2	2
Prospective collection of data	0	0	1	1	0	0	2	1	2	0	0	1	0	0
Appropriate endpoints	2	1	1	2	2	2	2	2	2	2	2	2	2	2
Unbiased assessment endpoints	0	0	0	0	2	0	0	0	0	0	2	0	0	0
Appropriate follow-up	2	1	2	0	2	2	2	2	2	2	2	2	2	1
Loss to follow-up < 5%	2	2	0	0	1	1	0	0	0	1	1	0	0	0
Prospective calculation study size	0	0	0	0	0	0	0	0	0	0	0	0	0	0
Adequate control group	-	-	-	-	-	-	-	0	-	-	-	-	1	-
Contemporary groups	-	-	-	-	-	-	-	2	-	-	-	-	2	-
Baseline equivalence of groups	-	-	-	-	-	-	-	2	-	-	-	-	1	-
Adequate statistical analysis	-	-	-	-	-	-	-	2	-	-	-	-	1	-
TOTAL	10	6	8	7	11	9	10	15	8	9	11	8	13	7

**Table 3:** Types of fracture in each study—GA, Gustilo–Anderson classification.

Author	Closed (%)	GA I (%)	GA II (%)	GA III A (%)	GA III B (%)	GA III C (%)
D.S. Jorgenson, 1995 [20]	0	0	0	0	3 (1.06)	0
H.S. Kim, 1998 [21]	0	0	0	0	1 (0.35)	0
G. Hosny, 2003 [22]	0	2 (0.7)	16 (5.65)	6 (2.12)	5 (1.77)	1 (0.35)
Ebrahim Hasankhani, 2006 [23]	0	0	0	19 (6.71)	9 (3.18)	4 (1.41)
David W. Lowenberg, 2013 [24]	2 (0.7)	0	2 (0.7)	7 (2.47)	20 (7.06)	3 (1.06)
J.P. Repo, 2016 [25]	3 (1.06)	0	0	1 (0.35)	5 (1.77)	7 (2.47)
Jia Xu, 2017 [26]	0	0	0	18 (6.36)		
Yongwei Wu, 2017 [27]	0	0	7 (2.47)	17 (6.00)	16 (5.65)	0
Mitsuhiko Takahashi, 2020 [28]	0	0	0	0	0	1 (0.35)
Abulaiti Abula, 2020 [29]	0	0	0	3 (1.06)	8 (2.83)	3 (1.06)
Jiang An Lim, 2020 [30]	0	3 (1.06)	6 (2.12)	4 (1.41)	7 (2.47)	0
Yong-Qing Xu, 2021 [31]	0	0	0	0	31 (10.95)	0
Christine M. Jones, 2021 [32]	0	0	0	0	17 (6.00)	1 (0.35)
Yuan-Jian Wu, 2022 [33]	0	0	0	25 (8.83)		

**Table 4:** Methods of reconstruction of the bone defects.

Author	Bone Graft	Compression—Distraction Osteogenesis	Bone Transport
D.S. Jorgenson, 1995 [20]	3	0	0
H.S. Kim, 1998 [21]	1	0	0
G. Hosny, 2003 [22]	0	0	4
Ebrahim Hasankhani, 2006 [23]	11	0	11
David W. Lowenberg, 2013 [24]	3	0	34
J.P. Repo, 2016 [25]	8	5	11
Jia Xu, 2017 [26]	0	2	10
Yongwei Wu, 2017 [27]	14	17	23
Mitsuhiko Takahashi, 2020 [28]	0	0	0
Abulaiti Abula, 2020 [29]	2	0	14
Jiang An Lim, 2020 [30]	4	0	4
Yong-Qing Xu, 2021 [31]	10	0	31
Christine M. Jones, 2021 [32]	0	14	0
Yuan-Jian Wu, 2022 [33]	0	25	0

**Table 5:** Types of reconstruction used for the soft tissue defects—X indicated the use of a specific type of reconstruction without mentioning the exact number of patients.

Author	Total Patients	Free Flap	Pedicle Flap	Local Flap	STSG	Other	Primary Closure
D.S. Jorgenson, 1995 [20]	3	2	2	0	0	0	0
H.S. Kim, 1998 [21]	1	1	0	0	0	0	0
G. Hosny, 2003 [22]	30	0	4	3	1	20	2
Ebrahim Hasankhani, 2006 [23]	32	X	0	X	X	0	X
David W. Lowenberg, 2013 [24]	34	33	0	2	0	0	0
J.P. Repo, 2016 [25]	16	16	0	0	0	0	0
Jia Xu, 2017 [26]	18	0	18	0	0	0	0
Yongwei Wu, 2017 [27]	40	8	12	3	4	0	0
Mitsuhiko Takahashi, 2020 [28]	1	1	0	0	1	0	0
Abulaiti Abula, 2020 [29]	14	3	11	0	0	0	0
Jiang An Lim, 2020 [30]	20	8	0	0	0	8	12
Yong-Qing Xu, 2021 [31]	31	0	0	0	9	22	0
Christine M. Jones, 2021 [32]	18	8	1	1	3	0	5
Yuan-Jian Wu, 2022 [33]	25	0	25	0	0	0	0

**Table 6:** End-points of the selected articles—NM, not mentioned.

Author	Total Patients	Union without Reintervention	Union after Reintervention	Flap Survival	Ambulation
D.S. Jorgenson, 1995 [20]	3	2	1	67%	100%
H.S. Kim, 1998 [21]	1	1	0	100%	100%
G. Hosny, 2003 [22]	30	30	0	100%	NM
Ebrahim Hasankhani, 2006 [23]	32	21	11	100%	69.75%
David W. Lowenberg, 2013 [24]	34	31	3	97.10%	97%
J.P. Repo, 2016 [25]	16	16	0	93.75%	100%
Jia Xu, 2017 [26]	18	6	10	100%	100%
Yongwei Wu, 2017	40	26	14	NM	NM
Mitsuhiko Takahashi, 2020 [28]	1	1	0	100%	100%
Abulaiti Abula, 2020 [29]	14	10	4	71.42%	100%
Jiang An Lim, 2020 [30]	20	19	0	NM	NM
Yong-Qing Xu, 2021 [31]	31	27	4	100%	100%
Christine M. Jones, 2021 [32]	18	18	0	92.85%	NM
Yuan-Jian Wu, 2022 [33]	25	20	5	100%	100%

**Table 7:** Complications described in every article in detail—NM, the exact number of patients was not mentioned. (%)—out of 283 patients.

Author	Total Patients	Superficial Infection/Pin Tract Infection	Deep Infection	Osteomyelitis	Deep Venous Thrombosis	Malunion	Refracture	Joint Stiffness—Knee	Joint Stiffness—Ankle	Limb Length Discrepancy	Pain	Flap Loss	Other
D.S. Jorgenson, 1995 [20]	3	0	0	0	0	0	0	0	0	0	0	1 (0.35)	1 (0.35)
H.S. Kim, 1998 [21]	1	0	0	0	0	0	0	0	0	1 (0.35)	0	0	1 (0.35)
G. Hosny, 2003 [22]	30	NM	0	0	2 (0.7)	2 (0.7)	0	2 (0.7)	0	2 (0.7)	0	0	3 (1.06)
Ebrahim Hasankhani, 2006 [23]	32	11 (3.89)	0	0	0	5 (1.77)	0	4 (1.41)	6 (2.12)	2 (0.7)	7 (2.47)	0	6 (2.12)
David W. Lowenberg, 2013 [24]	34	0	3 (1.06)	0	0	2 (0.7)	1 (0.35)	0	0	0	2 (0.7)	1 (0.35)	0
J.P. Repo, 2016 [25]	16	6 (2.12)	0	4 (1.41)	0	3 (1.06)	4 (1.41)	1 (0.35)	5 (1.77)	0	2 (0.7)	0	3 (1.06)
Jia Xu, 2017 [26]	18	5 (1.77)	0	0	0	0	0	0	0	0	0	0	4 (1.41)
Yongwei Wu, 2017 [27]	40	24 (8.48)	0	0	0	0	0	0	0	3 (1.06)	0	0	0
Mitsuhiko Takahashi, 2020 [28]	1	0	0	0	0	0	0	0	0	0	0	0	0
Abulaiti Abula, 2020 [29]	14	NM	0	0	0	NM	0	0	0	0	0	0	NM
Jiang An Lim, 2020 [30]	20	9 (3.18)	1 (0.35)	0	0	0	0	0	0	0	1 (0.35)	0	13 (4.59)
Yong-Qing Xu, 2021 [31]	31	2 (0.7)	0	3 (1.06)	0	2 (0.7)	1 (0.35)	7 (2.47)		0	0	0	0
Christine M. Jones, 2021 [32]	18	NM	NM	NM	NM	NM	NM	NM	NM	18 (6.36)	NM	1 (0.35)	NM
Yuan-Jian Wu, 2022 [33]	25	12 (4.24)	0	0	0	0	0	0	6 (2.12)	0	15 (5.3)	0	0

**Table 8:** Scores used to assess the patients’ evolution—ASAMI, Association for the Study and Application of the Method of Ilizarov classification.

**ASAMI Score**	**Bone Results**				**Functional Results**			
	**Excellent**	**Good**	**Fair**	**Poor**	**Excellent**	**Good**	**Fair**	**Poor**
Ebrahim Hasankhani, 2006 [23]	56.20%	12.50%	9.40%	21.80%	21.90%	43.80%	12.50%	21.80%
Abulaiti Abula, 2020 [29]	57.14%	42.86%	-	-	57.14%	42.86%	-	-
Yong-Qing Xu, 2021 [31]	19.35%	45.16%	25.80%	9.68%	25.80%	48.39%	16.13%	9.68%
**Paley’s Evaluation of Bone and Functional Results**	**Bone Results**				**Functional Results**			
	**Excellent**	**Good**	**Fair**	**Poor**	**Excellent**	**Good**	**Fair**	**Poor**
Yongwei Wu, 2017 [27]	78%	22%	-	-	36%	40%	24%	-
Yuan-jian Wu, 2022 [33]	72%	28%	-	-	48%	52%	-	-

## Data Availability

Data are contained within the article..
